# Exploratory insights into novel prehabilitative neuromuscular exercise-conditioning in total knee arthroplasty

**DOI:** 10.1186/s12891-022-05444-0

**Published:** 2022-06-07

**Authors:** Anna Maria Risso, Marietta L. van der Linden, Andrea Bailey, Peter Gallacher, Nigel Gleeson

**Affiliations:** 1 Centre for Health, Activity and Rehabilitation Research, Queen Margaret University Edinburgh, Edinburgh, EH21 6UU UK; 2 Robert Jones and Agnes Hunt Orthopaedic NHS Foundation Trust, Oswestry, SY10 7AG Shropshire UK

**Keywords:** Pre-surgery training, Total knee arthroplasty, Joint replacement, Sensorimotor, Neuromuscular, Exercise-conditioning

## Abstract

**Background:**

Contemporary strategies for prehabilitation and rehabilitation associated with total knee arthroplasty (TKA) surgery have focused on improving joint range-of-motion and function with less emphasis on neuromuscular performance beneficially affecting joint stability. Furthermore, prehabilitation protocols have been found to be too long and generic-in-effect to be considered suitable for routine clinical practice.

**Methods:**

A pragmatic exploratory controlled trial was designed to investigate the efficacy of a novel, acute prehabilitative neuromuscular exercise-conditioning (APNEC) in patients electing TKA. Adults electing unilateral TKA were assessed and randomly allocated to exercise-conditioning (APNEC, *n* = 15) and usual care (Control, *n* = 14) from a specialised orthopaedic hospital, in the United Kingdom. APNEC prescribed nine stressful exercise-conditioning sessions for the knee extensors of the surgery leg, accrued over one week (3 sessions·week^−1^; 36 exercise repetitions in total; machine, gravity-loaded) and directly compared with usual care (no exercise). Prescribed exercise stress ranged between 60%—100% of participant’s daily voluntary strength capacity, encompassing purposefully brief muscular activations (≤ 1.5 s). Baseline and follow-up indices of neuromuscular performance focusing on muscle activation capacity (electromechanical delay [EMD], rate of force development [RFD] and peak force [PF]) were measured ipsilaterally using dynamometry and concomitant surface electromyography (m. rectus femoris_[RF]_ and m. vastus lateralis_[VL]_).

**Results:**

Group mean ipsilateral knee extensor muscular activation capacity (EMD_RF_ [F_(3,57)_ = 53.5; *p* < 0.001]; EMD_VL_ [F_(3,57)_ = 50.0; *p* < 0.001]; RFD [F_(3,57)_ = 10.5; *p* < 0.001]) and strength (PF [F_(3,57)_ = 16.4; *p* < 0.001]) were significantly increased following APNEC (Cohen’s *d*, 0.5—1.8; 15% to 36% vs. baseline), but unchanged following no exercise control (*per protocol*, group *by* time interaction, factorial ANOVA, with repeated measures), with significant retention of gains at 1-week follow-up (*p* < 0.001).

**Conclusions:**

The exploratory APNEC protocol elicited significant and clinically-relevant improvement and its retention in neuromuscular performance in patients awaiting TKA.

**Trial registration:**

(date and number): clinicaltrial.gov: NCT03113032 (4/04/2017) and ISRCTN75779521 (3/5/2017).

## Background

Many recipients of total knee arthroplasty (TKA) are content with the outcome of surgery as an optimal solution for pain relief compared to physical rehabilitation. Exercise conditioning’s rationale is both intuitively and pragmatically appealing because preoperative enhancements to functional and fitness capabilities should lead to patients experiencing better surgical outcomes and reduced risks of further injury [1] and greater risk of falls [2, 3]. Amongst heterogeneous preoperative exercise methodologies and levels of evidential quality within meta-analytical reviews, significant improvements (small to moderate effects sizes) have been observed in postoperative function, quadriceps strength, and length of stay in patients undergoing TKA [4]. Similarly, while preoperative progressive resistance training (PRT) has been shown to be safe and not liable to exacerbate knee joint pain and effusion [5], the methodological quality of evidence had rendered the effectiveness of PRT before and/or after TKA as inconclusive [6]. Nevertheless, other reports support the use of short-term (4-week) high-intensity resistance training before TKA [7] and capable of inducing long-lasting effects on muscle strength [8], but not necessarily on functional performance [8] or other patient-reported outcomes [7]. Pronounced gains in functional, neuromuscular performance capabilities and higher patient satisfaction levels [9, 10] in related orthopaedic applications involving innovative and physiologically-principled resistance conditioning indicate the potential for improvement on contemporary approaches in which purposely-designed programmes can still have clear benefits for patients awaiting TKA.

Thus, while there is emerging evidence for the efficacy of pre-operative resistance training in contemporary literature for improving strength performance, we would argue that the exercise protocols that have been tested thus far [7, 8, 11, 12] may not have not been optimal with regard to their characteristics of physiological stress. As such, we hypothesise that a new approach incorporating ideals for neuromuscular conditioning may elicit more benefits.

Recent exploratory work in asymptomatic adults has shown efficacy within condensed, physiologically-principled approaches to conditioning [13]. As such, promoting short durations of muscle activation’ patterns during exercise in order to minimise the likelihood of pain nociceptor’ activation (< 1.5 s [14]) and unwanted provocation of autogenic and arthrogenic inhibition may be beneficial. Short durations of exercise conditioning may offer benefits in terms of efficacy, patients’ burdens and their adherence to prescribed programmes of exercise.

This paper reports on the exploratory findings of ipsilateral responses to acute prehabilitative neuromuscular exercise-conditioning (APNEC) in order to characterise its efficacy in patients awaiting TKA. It is part of a larger study investigating conditioning effects on both ipsilateral and contralateral limbs. Our hypothesis was that an acute and short dosage (one week, intermittently) of physiologically-principled exercise-conditioning, featuring high intensity (60% to 100% of daily strength capacity) and short durations of muscular activation, would show efficacy for improving neuromuscular performance. This study aimed to identify the efficacy of APNEC for improving neuromuscular performance capacities in patients awaiting TKA. Additionally, the retention of effects was explored at one week following cessation of APNEC.

## Methods

### Study design and participants

A UK single-centre NHS randomised controlled trial was registered (clinicaltrial.gov: NCT03113032 and ISRCTN75779521) and given Research & Development (RJAH/RL1 715) and ethical approval (South East Scotland Research Ethics Committee 01 [IRAS 198,930; REC reference 17/SS/0005]). The trial received endorsement by means of routine patient and public involvement meetings at the Robert Jones and Agnes Hunt (RJAH) Orthopaedic NHS Foundation Trust. Inclusion criteria involved patients over 18 years, diagnosed with severe OA of the knee and undergoing primary TKA surgery. Patients suffering from rheumatic or ongoing neurological disorders, conditions affecting function such as amputation, reduced mental capacity affecting abilities to follow prescribed exercises, or undergoing TKA for diseases other than OA, were excluded. Patients recording previous joint replacement surgery (contralateral knee, or hip) were not excluded.

The screening of the patients was undertaken within the hospital’s outpatient department by the clinicians having oversight (PG; AB). Inclusion criteria involved patients over 18 years, diagnosed with severe OA of the knee and undergoing primary TKA surgery. Patients gave written informed consent prior to baseline assessment (T1). Allocation to two groups (block randomisation [n, 2]) was performed subsequently by an independent statistician (randomization.com). Group allocation was not concealed from patients or from those overseeing the patients’ testing administration and conditioning within this explorative controlled trial.

The APNEC group (*n* = 15) underwent experimental intervention to the ipsilateral leg, receiving nine, 20-min focal exercise-sessions delivered on alternate days (3 sessions *per* week and 3 sessions *per* day) during a single week, with clinical oversight (AMR). The Control group (*n* = 14) followed current practice (no exercise).

### Assessment procedures and data capture

Participants were assessed ipsilaterally on four separate occasions that coincided with health care appointments for patients: T1 (baseline; ≈ 11 weeks prior to surgery, at initial clinical consultation, including familiarisation with assessment procedures and apparatus), T2 (pre-intervention; two weeks pre-surgery, at appointment for prophylactic infection control), T3 (48 h post-intervention; one week pre-surgery, immediate APNEC effects) and T4 (one week after cessation of intervention; retention of effects at TKA surgery).

### Acute prehabilitative neuromuscular exercise-conditioning (APNEC)

**Fig. 1:**
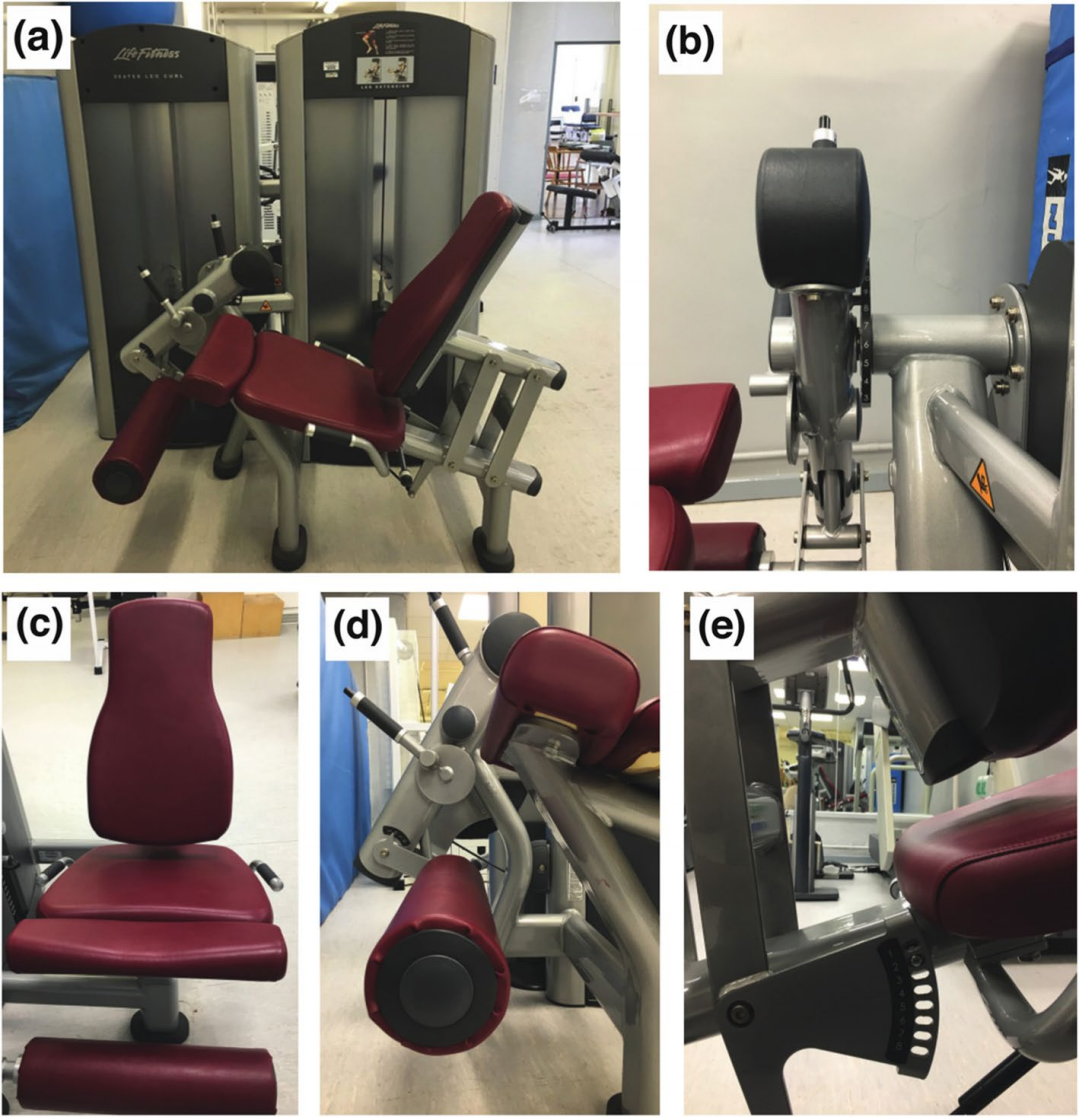
Knee extensor machine and settings **a** side view **b** knee position 1–10; this also helped secure the 90◦ starting position of the knee. **c** front view **d** padded leg rest setting: Small, Medium, Large and Extra large; this position determined where the padded leg rest will place pressure on the shin and was normally placed above the ankle **e** back seat rest setting 1–10; this was normally positioned so that the back of the knee rested comfortably over the machine’s seat-edge, with the shin hanging perpendicularly to the ground

**Fig. 2:**
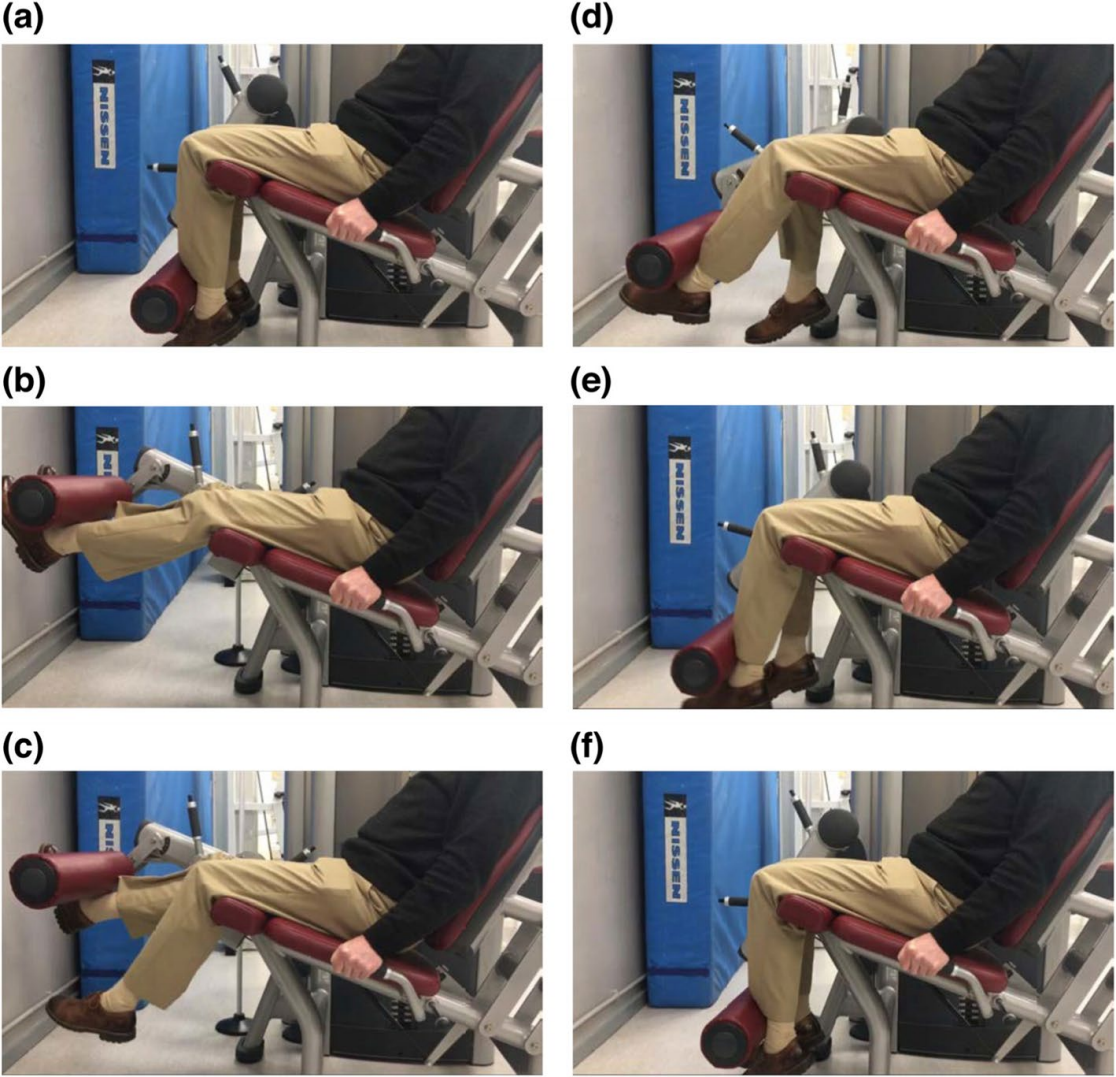
APNEC exercise movement. Please see main text for full explanation

APNEC prescribed nine stressful exercise-conditioning sessions for the ipsilateral knee extensors, accrued over one week (3 exercise days [24 h recovery]; 3 sessions *per* day [typically, 10.00 am; 1.00 pm; 4.00 pm]; 1 exercise *per* session; 4 repetitions *per* exercise, with 20 s inter-repetition rest periods [15] [36 repetitions in total] on a calibrated machine, gravity-loaded [Life Fitness, model number FZLE-500023; www.lifefitness.com] (Fig. 1a) and directly compared with usual care (no exercise). Prescribed exercise stress ranged between 60%—100% of participant’s daily voluntary strength capacity (one-repetition maximal [1RM], assessed using 2 or 3, 2-s concentric contractions and 15 min prior to first daily APNEC session) (Table 1), encompassing purposefully brief muscular activations (∼1.5 s). APNEC sessions were preceded by standardised warm-up (walk: ≈ 150 m, waiting area to gym) and recovery (240 s [16]), with an exercise repetition’ movements practised twice using an unloaded lever-arm (Fig. 1b, c, d and e), prior to exercise. Physiological stimuli for adaptation were delivered by micro-cyclical patterning of exercise intensity, recovery and progression, featuring high to low intra-day exercise intensity amongst daily sessions. Physiological stimuli for adaptation were delivered by micro-cyclical patterning of exercise intensity, recovery and progression, featuring high to low intra-day exercise intensity amongst daily sessions. The clinical model for APNEC had been derived and adapted from pilot research work involving micro- and meso-cyclical training theory and practices amongst elite professional soccer [17]. Figure 2 describes step-by-step, the performance of a single APNEC exercise repetition.

**Table 1:** An example of the micro-cyclical loading used during the APNEC delivery for participants randomised to this group. The nine sessions (S1—S9) reflect loading-progression delivered over a 1-week period (T2 to T3), with rest days interspersed amongst conditioning days

Session (S)	**Monday** (%MVC)	**Tuesday**		**Wednesday** (%MVC)	**Thursday**		**Friday** (%MVC)
S1	65%	Rest	S4	70%	Rest	S7	60%
S2	100%	Day	S5	90%	Day	S8	80%
S3	85%		S6	80%		S9	70%

Concentric muscle actions (patient self-paced) fully extended ipsi- and contralateral knees against the machine’s gravity-loaded lever-arm (Fig. 2a, start position). Bilateral full knee extension (Fig. 2b) momentarily preceded voluntary self-paced ipsilateral knee flexion to 45°, away from the lever-arm with maintained full contralateral knee extension (Fig. 2c). Contralateral knee extensor relaxation prompted the lever-arm’s unresisted gravitational loading (Fig. 2d). Ipsilateral extensor’s eccentric muscle action attempted to arrest and counteract the lever-arm’s downward trajectory very briefly (∼ 1.5 s) at ≈ 45° (Fig. 2e). A repetition was completed by the involved musculature’s relaxation and concomitant load-dropping to its starting position (Fig. 2f).

### Assessment of neuromuscular performance

Neuromuscular performance was assessed ipsilaterally focusing on muscle activation capacity (electromechanical delay [EMD]; rate of force development [RFD]) and knee extension strength (peak force [PF]) using dynamometry and concomitant surface electromyography (m. rectus femoris_[RF]_ and m. vastus lateralis_[VL]_). Participants were seated on a custom-built dynamometer, incorporating a load cell (range 3 kN; Tedea-Huntleigh, Cardiff, UK) [18, 19]. Knee and hip joint angles were standardised at 45° (0° representing full extension) [20] and 110° [21], respectively. Electromyographic (EMG) recordings were obtained using bipolar rectangular surface electrodes (self-adhesive, Ag/AgCl; 10 mm diameter; Unilect, UK), applied longitudinally and parallel to the muscle fibres’ orientation (30 mm inter-electrode distance [9, 18]; < 5 kΩ impedance [9, 22, 23]). EMG signals were pre-amplified (1902 Mk IV; Cambridge Electronic Design, UK; input impedance 10,000 MΩ, CMRR 100 dB gain of 1000) and filtered (Butterworth 2nd-order, low-pass, 1 kHz cut-off frequency).

Participants received an assessor’s verbal signal to activate (as rapidly and forcefully as possible) and relax their knee extensors (after 2 s to 3 s of activation), undertaking three static contractions separated by 10-s rest periods. Interrogation of raw EMG and concomitant force–time records (exceeding 95% confidence limits of the background electrical noise amplitude) [18, 19], identified peak performance of EMD_(RF and VL)_ (shortest latency [ms]), PF (highest force response [N]) and RFD (maximum rate at which force is increased (N·s^−1^) amongst the three trials. Prior to the assessment, participants warmed-up via subjectively-judged static contractions (~ 2 s; 10 s, inter-repetition neuromuscular recovery [15]) at 25% (2 repetitions), 50%, 75% and 100% of maximum voluntary contraction [18, 19].

### Statistical analysis

Group means (± SD) described outcome scores. Separate factorial (group [APNEC; Control] *x* time [T1; T2; T3; T4]) analyses of variance (ANOVAs), with repeated measures for time, tested hypotheses relating to EMD_RF_, EMD_VL_, RFD and PF using *per protocol* analyses (SPSS Vn. 23, IBM SPSS Illinois, USA). A priori reverse Helmert orthogonal difference testing located anticipated time-specific effects. Any violations of assumptions underpinning the use of ANOVA (for example, sampling from a normally distributed population, sphericity) were countered using Greenhouse–Geisser _(GG)_ adjustments. Statistical significance was accepted at *p* < 0.05, with Cohen’s *d* quantifying relative effect size (ES: 0.2, 0.5 and 0.8 considered in general to be ‘small’, ‘medium’ and ‘large’ effects, respectively) and percent change relative to ‘baseline’ performance offered as an additional marker of change in performance. A priori experimental design sensitivity estimation offered an approximate statistical power of 0.7 for avoiding intrusion of type-II errors for a medium relative effect size (Cohen’s *d*, 0.5) in the study’s primary outcome, EMD, and at its end-point (T4), requiring an approximate sample size of *n* = 12 within each group (www.sportsci.org).

## Results

**Fig. 3:**
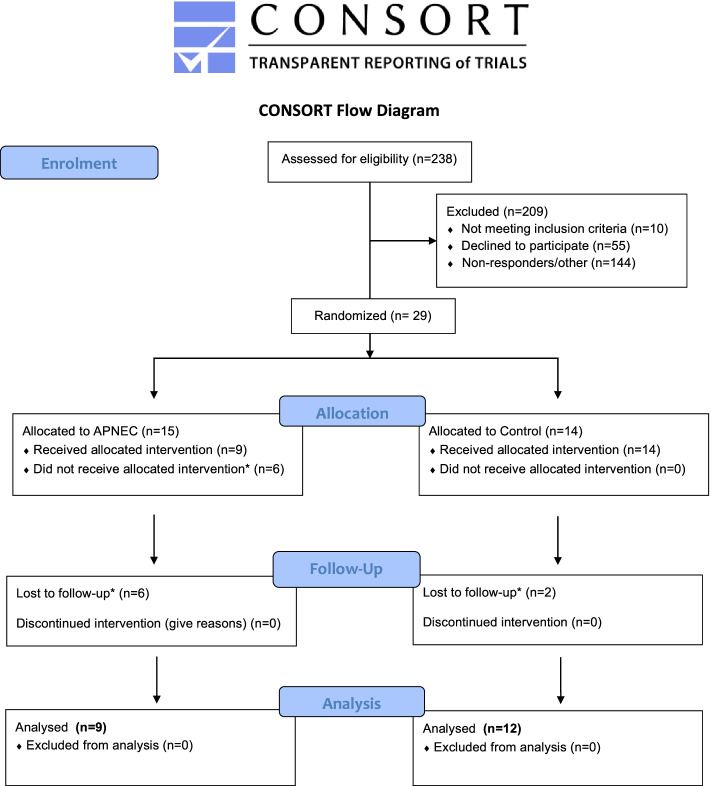
Study’s flow-chart of participants within the study based on the CONSORT guidelines for longitudinal studies

Twenty-nine patients from 238 candidates electing unilateral TKA (standardised implant procedure [MRK™]) on orthopaedic waiting-lists (May 2017 to April 2018) participated. Statistical analyses was undertaken on data from the 21 patients who completed the study protocol (Fig. 3: CONSORT enrolment; Table 2: *Per protocol* participants’ characteristics).

**Table 2:** *Per protocol* participants’ characteristics

**Characteristic**	**All** (*n* = 21)	**APNEC** (*n* = 9)	**Control** (*n* = 12)
Age (years)	71.1 ± 8.1	67.3 ± 6.1	74.0 ± 8.2
BMI (kg·m^−2^)	28.5 ± 10.4	26.8 ± 10.9	29.7 ± 9.9
Surgery waiting time (days)	83.4 ± 49.8	93.0 ± 54.6	82.8 ± 46.4
Right knee operated (number)	11.0	3.0	8.0
Previous arthroplasty (number)	7.0	1.0	6.0
MRK™ (number)	21.0	9.0	12.0

### Neuromuscular performance

**Fig. 4:**
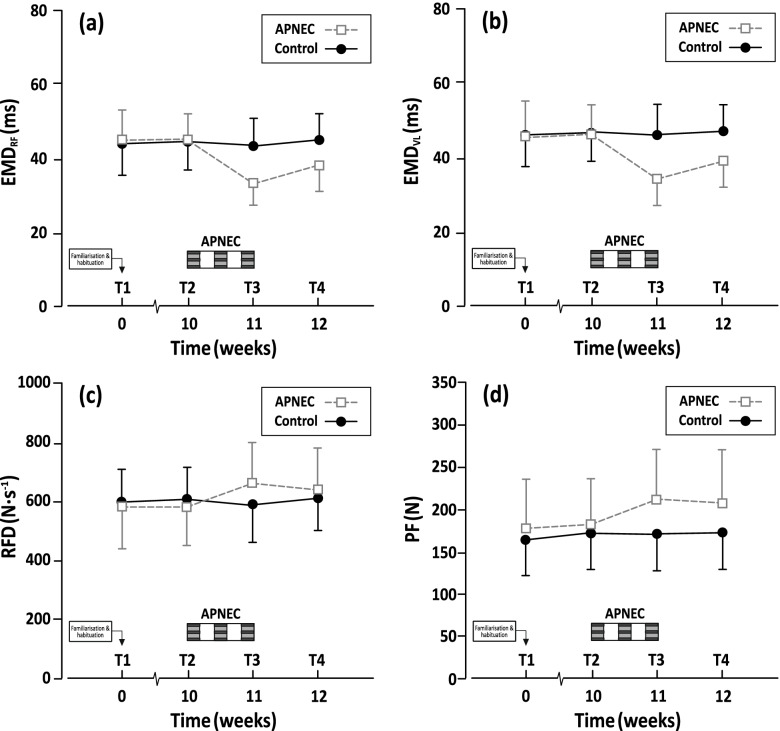
Group mean performance scores (± SD; APNEC, *n* = 9; Control, *n* = 12) assessed in the ipsilateral leg for muscle activation capacity (electromechanical delay [EMD]; rate of force development [RFD]) and knee extension strength (peak force [PF]) using dynamometry and concomitant surface electromyography (m. rectus femoris_[RF]_ and m. vastus lateralis_[VL]_)

Factorial interactions (group x time) showed group mean ipsilateral knee extensor muscular activation capacity (EMD_RF_ [F_(3,57)_ = 53.5; *p* < 0.0005]; EMD_VL_ [F_(3,57)_ = 50.0; *p* < 0.001]; and RFD [F_(3,57)_ = 10.5; *p* < 0.001]) and strength (PF [F_(3,57)_ = 16.4; *p* < 0.001]) were significantly increased immediately following APNEC, but unaffected by no exercise control (Table 3). Performance improvements between baseline and immediately after APNEC (EMD_RF_ [Cohen’s *d*, 0.62; 25.5%; F_(1,19)_ = 53.5; *p* < 0.0005, a priori difference contrast); EMD_VL_ [*d*, 0.66; 26.0%; F_(1,19)_ = 50.0; *p* < 0.0005]; RFD [*d*, 0.54; 13.2%; F_(1,19)_ = 10.5; *p* < 0.001]; PF [*d*, 0.55; 18.7%; F_(1,19)_ = 16.4; *p* < 0.001]) were prominent, contributing most to the overall ANOVA interactions, and were retained substantively at one week after APNEC’s cessation (T4: EMD_RF_ [Cohen’s *d*, 0.87; 14.7% vs. baseline; F_(1,19)_ = 26.5; *p* < 0.001, a priori difference contrast]; EMD_VL_ [*d*, 0.80; 15.0%; F_(1,19)_ = 26.8; *p* < 0.001]; RFD [*d*, 0.39; 9.5%; F_(1,19)_ = 8.2; *p* < 0.001]; PF [*d*, 0.46; 16.1%; F_(1,19)_ = 7.4; *p* < 0.001]) (Fig. 4).

**Table 3:** Group mean scores (± SD) for *per protocol* assessments at baseline (mean T1, T2), T3 and T4 for the leg undergoing surgery in the APNEC (*n* = 9, for all time points of assessment [5 males; 4 females]) and Control (*n* = 12, for all time points of assessment [7 males; 5 females]) groups. Calculated effect sizes (Cohen’s *d*) and percentage changes are relative to baseline performance

	**Baseline**	**T3**	**T4**	**ES**	**ES**
Outcome				Baseline – T3	Baseline – T4
**EMD**_**RF**_** (ms)**					
APNEC	45.3 ± 7.2	33.5 ± 6.0	38.4 ± 7.1	1.78	0.96
Control	45.0 ± 7.7	43.6 ± 7.5	45.3 ± 7.1	0.20	0.02
**EMD**_**VL**_** (ms)**					
APNEC	46.3 ± 8.1	33.9 ± 6.4	38.9 ± 8.3	1.70	0.91
Control	46.8 ± 8.1	45.3 ± 7.6	46.8 ± 7.4	0.20	0.01
**RFD (N·s**^**−1**^**)**					
APNEC	578.2 ± 127.2	658.7 ± 143.5	637.2 ± 145.7	0.59	0.43
Control	605.0 ± 114.8	587.2 ± 128.5	610.5 ± 134.0	0.10	0.01
**PF (N)**					
APNEC	182.4 ± 55.1	211.9 ± 62.8	207.1 ± 66.3	0.50	0.40
Control	171.8 ± 42.8	171.7 ± 44.9	172.8 ± 43.7	0.01	0.00

Key: *EMD* electromechanical delay (ms), *RF* rectus femoris muscle, *VL* vastus lateralis muscle, *RFD* rate of force development (N·s.^−1^), *PF* peak force (N)

## Discussion

This exploration study of prehabilitative neuromuscular exercise-conditioning in patients electing unilateral TKA showed the protocol’s efficacy for improving neuromuscular performance capacities, with substantial gains in peak ipsilateral knee extensor muscular activation characteristics (EMD [Cohen’s *d*, 0.62 – 0.66; 25.5 – 26.0% and RFD [*d*, 0.54; 13.2%]) and strength (PF [*d*, 0.55; 18.7%]) following APNEC. Concomitant responses of participants acting as controls were trivial (*d* < 0.2; < 3.0%; *ns*) and reflected contemporary practice in which no structured exercise was undertaken and only variations in activities of daily living might have elicited serendipitous conditioning stimuli.

Optimum dose–response characteristics for APNEC await scrutiny, but this formulation involving a short period of dosage, high intensity and purposely brief nociceptor response-evading muscular actions, elicited responses exceeding statistical, precision and reliability criteria for the selected indices of neuromuscular performance (≈ 4%—8%) [19, 20] and appears to offer important clinical relevance in counteracting persistent performance deficits [24, 25].

**Fig. 5:**
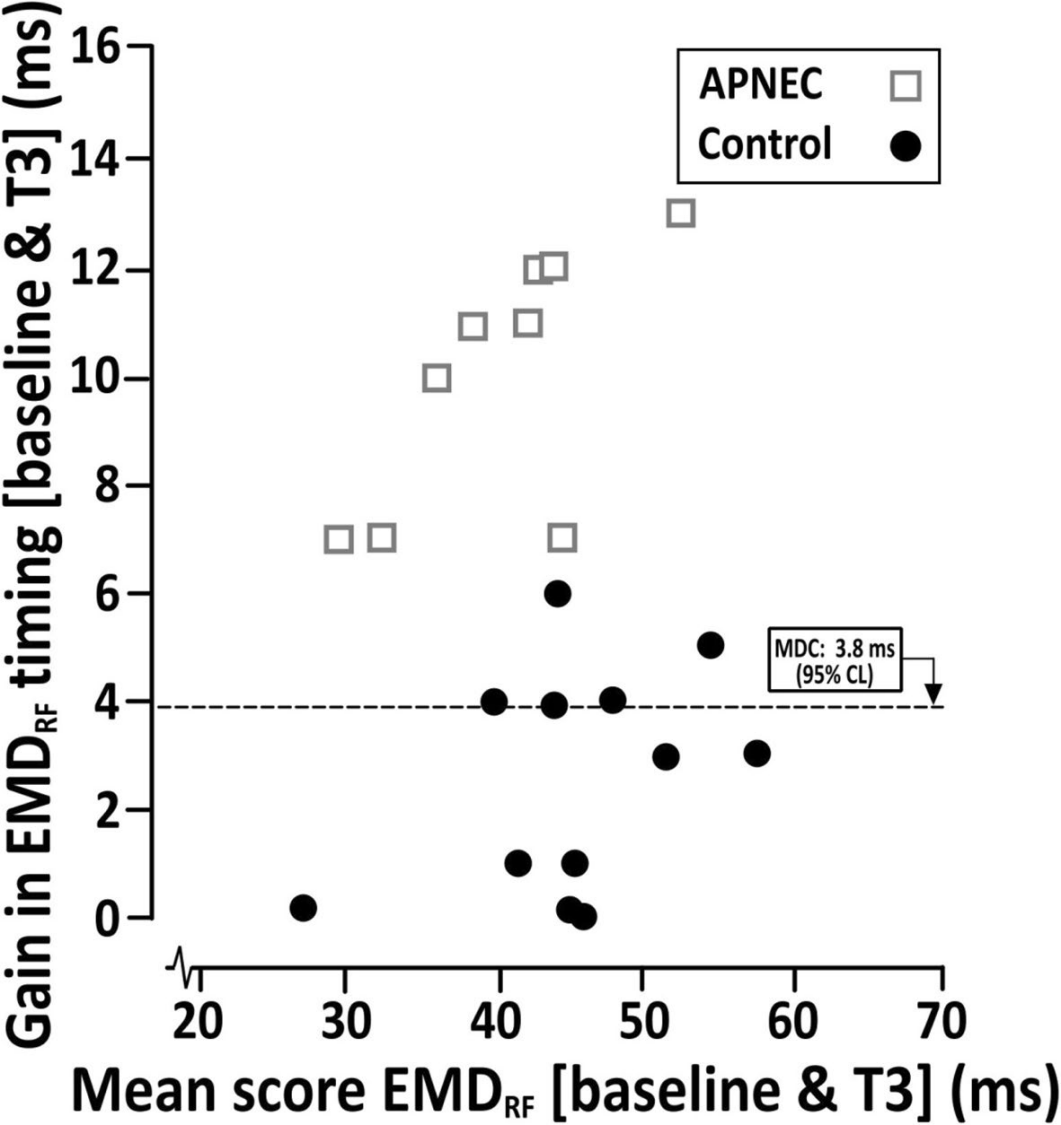
Patients’ individual improvement scores (APNEC, *n* = 9; Control, *n* = 12) for EMD (rectus femoris [_RF_]) of the ipsilateral leg (undergoing surgery), from baseline to the end of APNEC (absolute gain in EMD_RF_ performance [vertical axis: ms] plotted relative to the corresponding mean score associated with baseline and T3 performances [horizontal axis: ms]). Minimal detectable change associated with random measurement error in EMD (MDC; Estimated as an upper 95% confidence limit at 4.5% of pooled group mean scores: 3.8 ms) is superimposed for comparison

Relevant comparable potency data amongst the contemporary literature is elusive for all outcomes. Nevertheless, peak strength gains following APNEC (PF [*d*, 0.55]) matched or exceeded those amongst studies of established rehabilitative conditioning in patients undergoing TKA surgery [26, 27] or following conservative treatment [28, 29] (*d* ≤ 0.50). APNEC’s capability for improving several independent facets of neuromuscular performance (correlations amongst EMD, RFD and PF, at baseline: *r* < 0.40; *ns*) could be deployed usefully as a strategic alternative to aspects of contemporary prehabilitative practices associated with TKA, within end-stage OA in general, or serve as a specific augmentation. In the absence of definitive minimal clinically-important difference criteria [30], it was notable that gains for all APNEC patients exceeded the minimum detectable change criteria for EMD ([3.8 ms; 19) and the performance changes of all control patients (Fig. 5). Retention of gains in peak ipsilateral knee extensor muscular activation characteristics (EMD_RF_ [Cohen’s *d*, 0.91 – 0.96; 53.5%, *proportion of gain as a percentage*]; EMD_VL_ [Cohen’s *d*, 0.96; 53.9%] RFD [*d*, 0.43; 72.8%]) and strength (PF [*d*, 0.41; 80.0%]) following APNEC’s cessation were substantial, significantly better than baseline performance scores (*p* < 0.05) and had similarly exceeded the relevant minimum detectable change criteria.

There is emerging evidence for the efficacy of pre-operative resistance training in the contemporary literature for improving strength performance, including some protocols using relatively short durations (4 weeks) [7, 8, 11, 12]. By contrast, the efficacy of APNEC was derived from a more notably truncated protocol involving just nine stressful exercise-conditioning sessions for the ipsilateral knee extensors, accrued over one week, but encompassing deliberately brief muscular activations.

The APNEC protocol’s short duration emphases its potential for utility within clinical time-logistics. It also favours neural mechanisms rather than morphological adaptation [31] underpinning the gains in capabilities. There is neuromuscular performance capabilities. There is potential for APNEC’s characteristically brief, eccentric muscular actions to provoke favourable changes in neural activation patterns [32], with increased rates of motor unit firing [28]. Autogenic and arthrogenic inhibition may be reduced consequently by increased rates of motor firing, provoking sympathetic concomitant gains in neuromuscular performance [29, 32]. The complementarity of APNEC’s brief but intense muscle actions in mitigating nociceptor activation and potentiating increased activity amongst previously inhibited motor units may be crucial for efficacy in this clinical population. Purposely sequenced delivery of episodes of APNEC might feasibly offer potentiated effects on neuromuscular performance capacities in this clinical population, which could counter clinical criticisms of contemporary prehabilitative exercise programmes and their ability to elicit only subtle and transient improvements [33]. Future well-controlled clinical trials should continue to explore optimal modes and dose-responses of APNEC delivery in this population, which may occur in the absence of competing physiological conditioning stimuli, such as concomitant strength- and endurance-related exercise stresses [9].

Limitations to this study were related to its delivery and design. Logistical and ethical constraints had precluded assessments in the immediate period after surgery, and evaluation of longer-term decay patterning of APNEC gains in neuromuscular performance amongst complex interactive effects associated with surgery and post-TKA clinical care. Similarly, experimental controls were focused on an extended period of longitudinal evaluation of baseline performance capabilities of the leg undergoing surgery and differential inter-group responses, rather than on those of the contralateral leg.

Other limitations for this explorative trial included that group allocation was not concealed from patients or from those overseeing the patients’ testing administration and conditioning. As such, the potential for bias within the findings could not be eliminated. Similarly, physical activity behaviors associated with travel to and from the APNEC’s venue for its delivery and assessments was not monitored directly and varied physical activity might have elicited heterogeneous carry-over effects amongst the patients’ responses to APNEC. A further limitation reflected a lack of patients' self-perceived pain assessments within the APNEC protocol, even though the latter had been monitored in general by means of questionnaire, but not reported here. Furthermore, while the patient’s compliance with the APNEC’s training prescription was monitored directly, this approach that may not be facilitated in all environments, such as within self-managed care. Nevertheless, future studies will aim to identify optimised APNEC dosing and approaches for its scalability and delivery amongst varied care environments.

This study’s findings were derived from a modestly-sized sample of non-obese (BMI < 30 kg·m^−2^) patients (*n* = 21), which might preclude generalisation. Observed Type II error rates were modest (≤ 0.12) and appeared to offer suitable experimental design sensitivity and statistical power amongst the selected indices of neuromuscular performance.

## Conclusion

This exploration study of prehabilitative neuromuscular exercise-conditioning in patients electing unilateral TKA suggested that the APNEC protocol may be efficacious for improving neuromuscular performance capacities (*d*, 0.54—0.66; 13.6—26.0%). Gains prevailed the cessation of APNEC. 

## Data Availability

The datasets used and analysed during the current study are available from the corresponding author on reasonable request.

## References

[1] Lephart SM, Pincivero DM, Giraido JL, Fu FH (1997). The role of proprioception in the management and rehabilitation of athletic injuries. Am J Sports Med.

[2] Ratsepsoo M, Gapeyeva H, Vahtrik D, Aibast H, Ereline J, Haviko T (2011). Knee pain and postural stability in women with gonarthrosis before and six months after unilateral total knee replacement. Acta Kinesiol Univ Tartu.

[3] Bade MJ, Kohrt WM, Stevens-Lapsley JE (2010). Outcomes before and after total knee arthroplasty compared to healthy adults. J Orthop Sports Phys Ther.

[4] Moyer R, Ikert K, Long K, Marsh J (2017). The value of preoperative exercise and education for patients undergoing total hip and knee arthroplasty: a systematic review and meta-analysis. JBJS Rev.

[5] Skoffer B, Dalgas U, Maribo T, Søballe K, Mechlenburg I (2018). No exacerbation of knee joint pain and effusion following preoperative progressive resistance training in patients scheduled for total knee arthroplasty: secondary analyses from a randomized controlled Trial. J PRMJ.

[6] Skoffer B, Dalgas U, Mechlenburg I (2015). Progressive resistance training before and after total hip and knee arthroplasty: a systematic review. Clin Rehabil.

[7] Skoffer B, Maribo T, Mechlenburg I, Hansen PM, Søballe K, Dalgas U (2016). Efficacy of preoperative progressive resistance training on postoperative outcomes in patients undergoing total knee arthroplasty. Arthritis Care Res.

[8] Skoffer B, Maribo T, Mechlenburg I, Gaarden Korsgaard C, Søballe K, Dalgas U (2020). Efficacy of preoperative progressive resistance training in patients undergoing total knee arthroplasty: 12-month follow-up data from a randomized controlled trial. Clin Rehabil.

[9] Bailey A, Minshull C, Richardson J, Gleeson N (2014). Non-concurrent strength and cardio-vascular endurance rehabilitation conditioning improves outcome following ACI surgery to the knee. J Sport Rehabil.

[10] Moutzouri M, Gleeson N, Coutts F, Tsepis E, Gliatis I (2018). Does enhanced sensorimotor training affect functional and balance performance on patients following total knee replacement? A single-blind randomised controlled trial. Clin Rehabil.

[11] Huber EO, Roos EM, Meichtry A, de Bie RA, Bischoff-Ferrari HA (2015). Effect of preoperative neuromuscular training (NEMEX-TJR) on functional outcome after total knee replacement: an assessor-blinded randomized controlled trial. BMC Musculoskelet Disord.

[12] Pohl T, Brauner T, Wearing S, Stamer K, Horstmann T (2015). Effects of sensorimotor training volume on recovery of sensorimotor function in patients following lower limb arthroplasty. BMC Musculoskelet Disord.

[13] Peer MA, Gleeson N (2018). Effects of ashort-term conditioning intervention on knee flexor sensorimotor and neuromuscular performance in men. J Sport Rehabil.

[14] Fein A (2012). Nociceptors and the perception of pain. Univ Conn Health Center.

[15] Moore MA, Kukulka CG (1991). Depression of Hoffmann reflexes following voluntary contraction and implications for proprioceptive neuromuscular facilitation therapy. Phys Ther.

[16] Jamurtas AZ, Fatouros IG, Buckenmeyer P, Kokkinidis E, Taxildaris K, Kambas A (2000). Effects of plyometric exercise on muscle soreness and plasma creatine kinase levels and its comparison with eccentric and concentric exercise. J Strength Cond Res.

[17] Clancy C, Gleeson N, Mercer T (2021). Neuromuscular performance and training workload over an in-season mesocycle in elite young soccer players. Int J Sports Physiol Perform.

[18] Minshull C, Gleeson N, Walters-Edwards M, Eston R, Rees D (2007). Effects of acute fatigue on the volitional and magnetically-evoked electromechanical delay of the knee flexors in males and females. Eur J Appl Physiol.

[19] Minshull C, Gleeson N, Eston R, Bailey A, Rees D (2009). Single measurement reliability and reproducibility of volitional and magnetically-evoked indices of neuromuscular performance in adults. J Electromyogr Kinesiol.

[20] Gleeson N, Naish P, Wilcock J, Mercer T (2002). Reliability of indices of neuromuscular leg performance in end-stage renal failure. J Rehabil Med.

[21] Vahtrik D, Gapeyeva H, Aibast H, Ereline J, Kums T, Haviko T (2012). Quadriceps femoris muscle function prior and after total knee arthroplasty in women with knee osteoarthritis. Knee Surg Sports Traumatol Arthrosc.

[22] Mercer T, Gleeson N, Claridge S, Clement S (1998). Prolonged intermittent high intensity exercise impairs neuromuscular performance of the knee flexors. Eur J Appl Physiol.

[23] Gleeson N, Eston R, Reilly T (2018). Assessment of neuromuscular performance using electromyography. Kinanthropometry and Exercise Physiology Laboratory Manual: Tests, Procedures and Data, Volume 2: Exercise Physiology (4th Edition).

[24] Valtonen A, Poyhonen T, Heinonen A, Sipila S (2009). Muscle deficits persist after unilateral knee replacement and have implications for rehabilitation. Phys Ther.

[25] Maffiuletti NA, Bizzini M, Widler K, Munzinger U (2010). Asymmetry in quadriceps rate of force development as a functional outcome measure in TKA. Clin Orthop Relat Res.

[26] Swank AM, Kachelman JB, Bibeau W, Quesada PM, Nyland J, Malkani A (2011). Prehabilitation before total knee arthroplasty increases strength and function in older adults with severe osteoarthritis. J Strength Cond Res.

[27] McKay C, Prapavessis H, Doherty T (2012). The effect of a prehabilitation exercise program on quadriceps strength for patients undergoing total knee arthroplasty: A randomized controlled pilot study. Phys Med Rehabil J.

[28] Van Cutsem M, Duchateau J, Hainaut K (1998). Changes in single motor unit behaviour contribute to the increase in contraction speed after dynamic training in humans. J Physiol.

[29] Aagaard P, Simonsen E, Andersen J, Magnusson S, Halkjaer-Kristensen J, Dyhre-Poulsen P (2000). Neural inhibition during maximal eccentric and concentric quadriceps contraction: effects of resistance training. J Appl Physiol.

[30] Keurentjes J, Van Tol F, Fiocco M, Schoones J, Nelissen R (2012). Minimal clinically important differences in health-related quality of life after total hip or knee replacement: a systematic review. Bone Joint Res.

[31] Suetta C, Aagaard P, Rosted A, Jakobsen AK, Duus B, Kjaer M (2004). Training-induced changes in muscle CSA, muscle strength, EMG, and rate of force development in elderly subjects after long-term unilateral disuse. J Appl Physiol.

[32] Suchomel TJ, Nimphius S, Bellon CR, Stone MH. The importance of muscular strength: training considerations. Sports medicine. 2018;48(4):765–85. 10.1007/s40279-018-0862-z 29372481

[33] Wang L, Lee M, Zhang Z, Moodie J, Cheng D, Martin J (2016). Does preoperative rehabilitation for patients planning to undergo joint replacement surgery improve outcomes? A systematic review and meta-analysis of randomised controlled trials. BMJ.

